# Construction and Analysis of the Physical Fitness Evaluation Index System for Elite Male Singles Badminton Players: Based on Delphi and AHP Methods

**DOI:** 10.3390/life14080944

**Published:** 2024-07-28

**Authors:** Binyong Ye, Houwei Zhu, Zhen Yang, Zhanyang He, Gongju Liu, Huiju Pan, Haiying Guo

**Affiliations:** 1 College of Physical Education and Health Sciences, Zhejiang Normal University, Jinhua 321000, China; binyongye@zjnu.edu.cn (B.Y.); zhuhouwei@zjnu.edu.cn (H.Z.); yz19550596533@163.com (Z.Y.); 2908161568@zjnu.edu.cn (Z.H.); panhuiju@zjnu.cn (H.P.); 2 Laboratory of Aquatic Sports Science of General Administration of Sports China, Zhejiang College of Sports, Hangzhou 311200, China; liugongju@hotmail.com

**Keywords:** elite athlete assessment, sports performance indicators, performance measurement, assessment indicators

## Abstract

Objective: To construct and validate a physical fitness evaluation index system for elite male singles badminton players. Methods: Utilizing the Delphi method to establish a comprehensive evaluation system, the analytic hierarchy process (AHP) was employed to calculate the influence weights of various indicators. The validity of the comprehensive evaluation system was verified using testing methods. Results: After three rounds of expert selection, the physical fitness evaluation index system for elite male singles badminton players includes three primary indicators, nine secondary indicators, and twenty-one tertiary indicators. Among the primary indicators, specialized physical fitness holds a significant weight in the evaluation with a value of 0.651, whereas body morphology has a smaller weight of 0.077. Among the secondary indicators, specialized agility, strength, and endurance have higher weights of 0.223, 0.217, and 0.210, respectively. Among the tertiary indicators, four-corner ball touch, 400 m × 5 shuttle run, smash-and-rush, and vertical jump height hold higher weights of 0.119, 0.114, 0.104, and 0.096, respectively. The results after randomly selecting ten elite male singles badminton players and applying the evaluation index system demonstrated that this system has high feasibility and validity. It can not only comprehensively assess the physical fitness of athletes but also provide significant practical guidance for enhancing their competitive performance. Conclusions: The evaluation system and weight assignments constructed in this study can scientifically and comprehensively reflect the physical fitness status of athletes. It can guide coaches in formulating targeted training plans and optimizing training outcomes.

## 1. Introduction

As a globally popular sport, badminton has numerous enthusiasts and outstanding athletes in China. Since the change to the 21-point scoring system in 2006, the unpredictability of match results has significantly increased, greatly enhancing the intensity and watchability of the games. Under the new scoring system, the pace of the game is faster, and rallies are more frequent, requiring athletes to start over 500 times and cover a distance of more than 3000 m in a single match [1]. These changes place unprecedented physical demands on athletes. Players need not only exceptional explosive power and speed but also the ability to maintain consistent performance during prolonged high-intensity competition, challenging their endurance, recovery ability, and physical reserves [2,3]. Physical fitness is considered the ability of an individual to perform muscle work satisfactorily, which requires a good level of cardiovascular function, muscular strength, speed, endurance, agility, and other attributes. Physical fitness often becomes the key factor in determining victory, especially in critical stages of competition. The coordinated development of these attributes not only enhances individual athletic performance but also improves adaptability and stress resistance in daily life [4]. The importance of physical fitness is evident not only in sports competitions but also as an indispensable part of our daily lives. Therefore, the systematic and scientific nature of physical training is particularly important in modern badminton [5]. Through scientific physical training, athletes can not only enhance their competitive level and technical stability but also effectively prevent sports injuries, thereby enabling them to stand out in intense competitions and achieve excellent results [6].

As a key competitive sport and a major gold-winning event in China, badminton boasts numerous outstanding players. However, how to evaluate the physical fitness of athletes scientifically and objectively and develop personalized training programs remains an urgent issue to be addressed. An analysis of existing literature reveals current research on physical fitness evaluation systems in badminton focuses mainly on young athletes, talent selection, and certain special groups [7,8,9]. Some studies have primarily focused on specific physical fitness indicators, such as speed and strength, while neglecting other critical factors [10,11]. Additionally, certain studies have not employed scientific methodologies, such as the Analytic Hierarchy Process, to establish a systematic and objective evaluation indicator system. Instead, they have relied excessively on the researchers’ personal experience and intuition, potentially leading to results with a high degree of subjectivity [12]. Research on the physical fitness evaluation system for high-level adult male badminton players has also been lacking, resulting in a failure to meet the needs of practical training fully. Additionally, existing research generally lacks practical application verification of the constructed evaluation systems, thus failing to sufficiently demonstrate their scientific validity and feasibility, making it difficult to ensure that these systems have practical application.

In the absence of practical evidence or recommendations, individual experience becomes the practitioner’s alternative. As Minas et al. [13] stated, although the final result of expert consultation may be incorrect, consensus usually provides a better basis than individual judgment. Therefore, in the absence of direct evidence, the Delphi method provides a scientific decision-making tool to ensure scientific validity and reliability [14]. The Delphi method is widely used in establishing evaluation systems and determining indicators. It systematically collects and synthesizes opinions through repeated consultations with a group of independent experts, aiming to achieve consensus and predictions, ultimately forming more objective and comprehensive conclusions [15]. However, while the Delphi method significantly reduces individual bias through expert consensus, its results are often qualitative, difficult to quantify directly, and may carry subjectivity. It is suitable for identifying key issues and forming preliminary plans but may lack precision and objectivity in practical application [16,17]. Some studies have followed the Delphi method with the analytic hierarchy process (AHP) to address these shortcomings and further refine and quantify expert opinions, thereby enhancing the scientific validity and reliability of decisions. However, these studies often do not apply the constructed indicator systems in practice, which may undermine their scientific validity and reliability, ultimately affecting the practical significance and application effectiveness of the indicator systems.

In summary, this study aims to construct a physical fitness evaluation index system for elite male singles badminton players using the Delphi method and the AHP. In the final stage of the study, high-level athletes were selected randomly to apply and validate the established index system. Through the scientific and rational establishment and application of the index system, this study provides a theoretical basis for the comprehensive evaluation of athletes’ physical fitness and has important practical significance for improving their competitive performance.

Research Hypothesis: Through several rounds of expert consultation and feedback, the Delphi Method can establish a comprehensive and scientific physical fitness evaluation index system. By employing the Analytic Hierarchy Process (AHP), the weights of various indicators can be calculated to determine their relative importance in the overall evaluation. The application of this comprehensive evaluation system can accurately assess the physical fitness of elite male singles badminton players, thereby guiding coaches in formulating targeted training plans, optimizing training outcomes, and enhancing the competitive performance of athletes.

## 2. Research Process and Methods

### 2.1. Research Process

**Figure 1:**
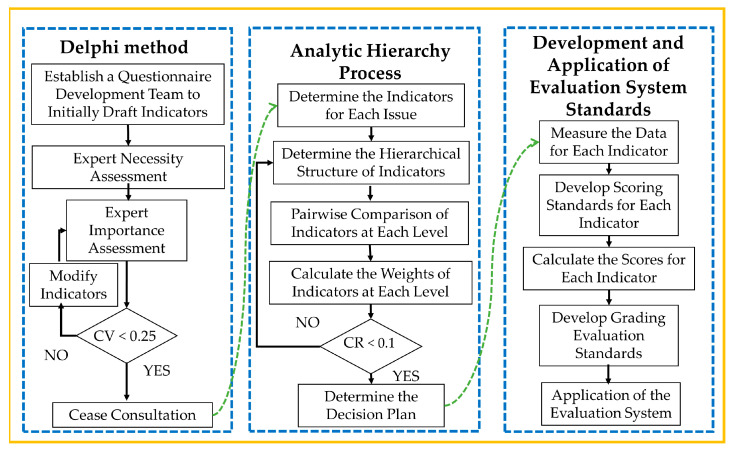
Research Process.

The research process of this study is illustrated in Figure 1. First, the Delphi method was employed to invite experts to evaluate the necessity and importance of the preliminary-drafted indicators. Consultation ceased when expert opinions tended to converge and the establishment of the evaluation index system was completed. Subsequently, a hierarchical structure model was constructed. The AHP was then used to address the qualitative and quantitative analyses of each indicator, providing an in-depth analysis of the complex decision-making problem from the perspectives of the essence of the problem, influencing factors, and their intrinsic relationships. This approach aims to offer concise and clear decision-making solutions. Finally, the completed decision-making plan was applied in practice to verify and support these evaluation system standards further.

### 2.2. Research Methods

#### Preliminary Establishment of Evaluation Indicators

This study systematically analyzed recent literature, works, and policy documents concerning badminton physical training, specialized physical fitness, physical attributes, and the physical fitness measurement and evaluation standards established by the General Administration of Sport of China. Additionally, in-depth discussions and detailed records were conducted with high-level badminton players, coaches, referees, and related personnel regarding influencing factors and other relevant issues. Then, the three basic dimensions were preliminarily identified, including the body morphology, basic physical fitness, and specialized physical fitness of badminton players. Based on these dimensions, a consultation questionnaire was prepared, initially establishing three primary indicators.

**Figure 2:**
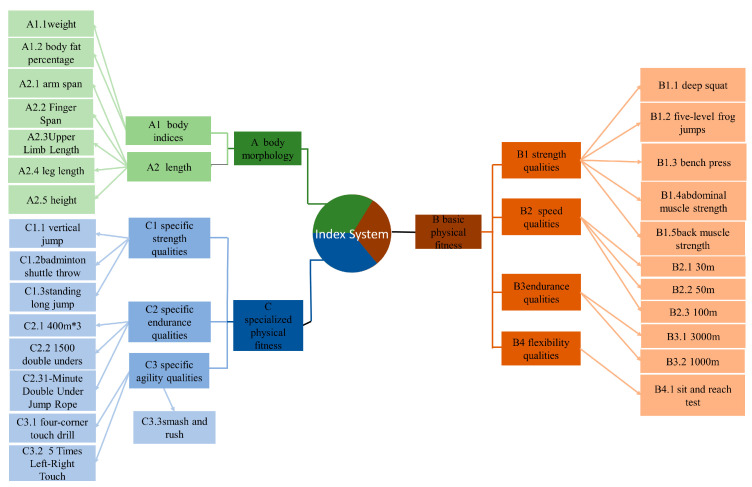
Preliminary indicators of the physical fitness evaluation system for elite male singles badminton players.

A questionnaire preparation team of eight members was formed to ensure the validity, objectivity, comprehensiveness, and comparability of the physical fitness evaluation index system consultation questionnaire. This team included two coaches with over 30 years of coaching experience, two researchers with over 20 years of research experience, one national-level coach with over 10 years of coaching and research experience, two associate professors, and one lecturer. Following the fundamental value orientations of contributing to the development of national sports, guiding scientific training for coaches, and promoting the personal quality improvement of athletes, after multiple discussions, the first round of the consultation questionnaire was determined to include three primary indicators, nine secondary indicators, and twenty-seven tertiary indicators (Figure 2) [9,18,19,20,21,22,23,24,25,26,27,28].

### 2.3. Delphi Expert Consultation Method

#### 2.3.1. Expert Selection Criteria, Expert Positive Coefficient, and Authority Coefficient

This study invited a total of 15 experts from both industry and academia to participate in the Delphi method consultation to ensure the representativeness and comprehensiveness of the expert consultation. The selection of experts was based on the following basic criteria: 1. The expert must have at least ten years of relevant research experience, especially a solid theoretical and practical background in sports science and physical fitness evaluation. 2. The expert must have extensive coaching experience, with a minimum of five years of coaching high-level sports teams and guiding athletes to achieve excellent results in provincial or national competitions. All experts voluntarily participated in the study and committed to completing at least two rounds of consultation. The basic information about the expert group is detailed in Table 1.

The expert positive coefficient is expressed by the questionnaire recovery rate, reflecting the degree of attention experts pay to this study. Typically, a recovery rate greater than 70% indicates high expert enthusiasm [29]. The expert authority coefficient (Cr) is calculated through statistical analysis based on the expert’s judgment basis (Ca) and degree of familiarity (Cs), using the formula Cr = (Ca + Cs)/2. When CS ≥ 1, it indicates “Very Familiar”; for 1 > CS ≥ 0.8, it denotes “Fairly Familiar”; for 0.8 > CS ≥ 0.5, it signifies “Familiar”; for 0.5 > CS ≥ 0.3, it represents “Slightly Familiar”; and for 0.3 > CS ≥ 0.1, it is described as “Not Familiar”. The range of Cr is usually between 0 and 1, with higher values indicating higher authority among the participating experts and greater reliability of the consultation results. Generally, a Cr ≥ 0.70 is considered acceptable [7,30]. The values assigned to Ca are detailed in Table 2.

#### 2.3.2. Expert Consultation Process

Step 1: Consultation on the necessity of physical fitness evaluation indicators for elite male singles badminton players. Experts were asked to assess the necessity of three primary indicators, nine secondary indicators, and 27 tertiary indicators through the options “Select”, “Delete”, and “Modify”. Step 2: Incorporate the modifications suggested by the experts in the first round to form a new physical fitness evaluation index system for elite male singles badminton players. Experts were then invited to rate the importance of the new index system on a five-point scale: “Very Important”, “Important”, “Moderately Important”, “Less Important” and “Not Important”.

#### 2.3.3. Indicator Screening Criteria

The number of evaluation criteria is not necessarily the more, the better; the focus should be on the significance of each criterion’s role in the evaluation. In the initial stage of establishing the evaluation index system, a large amount of redundant information may affect the accuracy of the evaluation results. Thus, to further ensure the accuracy of the evaluation results, a degree of expert opinion coordination was introduced to screen the value of the indicators. The degree of expert opinion coordination reflects the consistency of consulting experts’ judgments on various indicators. It is represented by the coefficient of variation (CV, the standard deviation of each indicator divided by its mean value) and the Kendall coordination coefficient (W) [29]. A smaller CV indicates that experts’ opinions on a particular indicator tend to be consistent, with a CV < 0.25 being acceptable. The range of W is between 0 and 1, with values closer to 1 indicating better coordination among experts on the ratings of all factors. Generally, a Kendall coordination coefficient < 0.2 indicates poor consistency; 0.2–0.4 indicates moderate consistency; 0.4–0.6 indicates medium consistency; 0.6–0.8 indicates strong consistency; and 0.8–1.0 indicates very strong consistency. This study uses the mean score of experts’ importance ratings for each indicator, CV and W, as the screening criteria. An indicator passes the screening, and consultation stops if the mean score > 3.5, CV < 0.25, W is between 0.4 and 0.5, and the asymptotic significance *p* < 0.05 [31,32,33].

### 2.4. Analytic Hierarchy Process

The AHP is a commonly used method for quantifying qualitative issues and is widely applied in studies involving the establishment of indicator systems [34,35]. Its application in assigning weights to indicators involves establishing an ordered hierarchy and comprehensively calculating the weight coefficients of the indicators by comparing the relative importance of each indicator within the same level [36,37].

**Figure 3:**
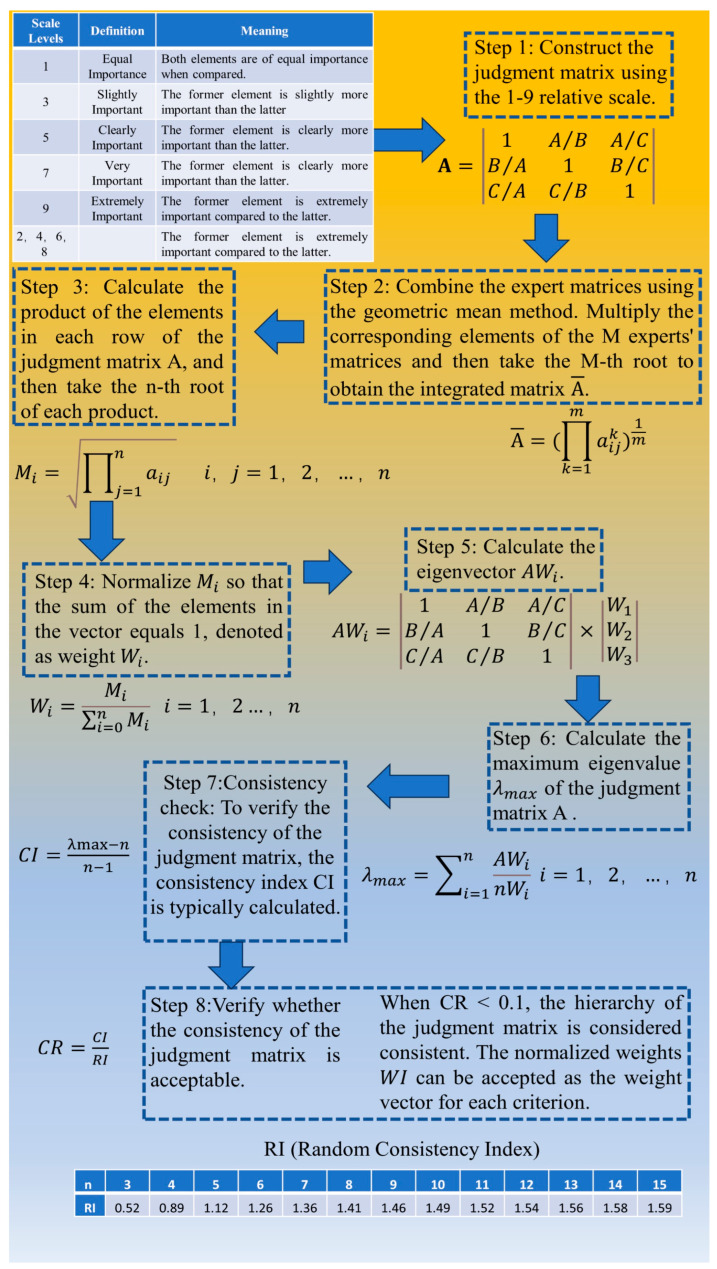
Calculation process of analytic hierarchy process.

The AHP establishes a hierarchical structure model composed of the overall goal, criteria, and candidates, each of which is independently unaffected by the others. Based on different evaluation factors, candidates are compared pairwise according to the 1–9 relative ranking scale provided by Saaty to form a comparative judgment matrix. The scoring matrices of all experts are combined into a judgment integration matrix using the geometric mean method, and the weight value of each candidate relative to the overall goal is calculated [38,39]. Through this process, the relative importance of each indicator at each level is judged, thus quantifying subjective evaluations and systematically assessing the importance weights of each indicator relative to the overall goal. The specific calculation process can be seen in Figure 3.

Note: Step 1: A (judgment matrix); A, B, C (element); A/B (relative importance of element A compared with element B).

Step 2: $\bar{A}$ (combined matrix); $a_{ij}^{k}$ (element in the *i*-th row and *j*-th column of the *k*-th expert’s judgment matrix); *m* (number of experts).

Step 3: $M_{i}$ (*n*-th root of the product of elements in the *i*-th row); *n* (dimension of the matrix); $a_{ij}$ (element in the *i*-th row and *j*-th column of the judgment matrix).

Step 4: $W_{i}$ (normalized weight of the *i*-th element); $\sum_{i=0}^{n}M_{i}$ (sum of the products of all elements).

Step 5: ${AW}_{i}$ (eigenvector); $W_{i}$ (normalized weight of the *i*-th element).

Step 6: $\lambda_{max}$ (maximum eigenvalue of the judgment matrix); ${AW}_{i}$ (eigenvector); ${nW}_{i}$ (product of the matrix dimension and the normalized weight).

Step 7: CI (consistency Index); $\lambda_{max}$ (maximum eigenvalue of the judgment matrix); *n* (dimension of the matrix).

Step 8: CR (consistency ratio); CI (consistency index); RI (random consistency index).

### 2.5. Testing Method

This study was conducted in two phases. In the first phase, 15 elite male singles badminton players were randomly selected to test predefined indicators. The mean, standard deviation, maximum, and minimum values for each indicator were calculated to establish a general value model for the physical fitness of elite male badminton players. Scoring standards were then developed to assign accurate numerical values to specific evaluation criteria, ensuring that the results are fair, objective, and quantifiable. To verify the independence of the evaluation index system and reduce potential sample bias, the second phase of the study employed a different sample group. An additional 10 players, who were distinct from the initial 15 and varied in their abilities and training backgrounds, were tested. This approach was taken to confirm the universality and effectiveness of the evaluation system.

The inclusion criteria for participants were as follows: age between 18 and 35 years, no injuries in the past year, at least three years of professional training experience, and outstanding performance in various major competitions. This stringent selection process ensured that the participants were representative of elite badminton players, thus providing robust data for validating the evaluation system. All tests were conducted under standardized conditions using calibrated equipment to ensure consistency and safety. Participants received detailed instructions and demonstrations to ensure proper execution of each test. Professional supervisors were present throughout the testing process to oversee procedures and ensure accurate data recording. This study was approved by the Ethics Committee of Zhejiang Normal University (No. ZSRT2024165). Participants were informed about the experimental procedures and provided written informed consent prior to the experiment. The study was conducted in accordance with the principles of the Declaration of Helsinki, with all methods and procedures designed to uphold ethical standards.

## 3. Results 

### 3.1. Statistical Analysis of Experts’ Basic Information

In this study, a survey was conducted with 15 experts from industry and academia. The survey included information on the experts’ age, years of work experience, and profession. All 15 experts participated in the first round of the survey. The survey results indicate that these experts possess high authority. The results indicate the expert group has substantial work experience and high educational backgrounds, providing a certain level of representativeness and rationality, which ensures the content authenticity and structural rationality of the constructed evaluation index system.

Regarding expert enthusiasm, this study conducted four rounds of expert consultations, with a 100% questionnaire recovery rate for each round. The questionnaire recovery rate is typically used to calculate the expert positive coefficient, and a recovery rate above 70% indicates high expert enthusiasm [29]. Therefore, the 15 experts consulted in this study showed high levels of support and assistance, giving the survey results high reliability and application value. Additionally, based on the quantitative method of experts’ judgment and familiarity, the authority coefficient of the first round of experts was calculated. The values were Ca = 0.892 and Cs = 0.73, with Cr values ranging between 0.7 and 1.0 and a mean value of 0.826, indicating that the experts consulted in this study have high authority, further ensuring the scientific validity and authority of the research results.

### 3.2. Results of the First Round of Expert Consultation

The purpose of the first round of expert consultation was to assess the necessity of the initially drafted three primary indicators, nine secondary indicators, and 27 tertiary indicators using the options “Select”, “Delete” and “Modify”, with a consensus threshold set at 70% [40]. The results of the expert consultation indicated that no modifications, deletions, or additions were needed for the primary and secondary indicators. However, for the tertiary indicators, “Finger Span”, “Upper Limb Length”, “100 m Run” and “5 Times Left-Right Touch” were deleted. Additionally, “Smash and Rush” was modified to “Smash and Net Kill”. 

### 3.3. Results of the Second Round of Expert Consultation

Incorporating the modifications suggested by experts in the first round, a new physical fitness evaluation index system for elite male singles badminton players was formed after qualitative screening of the indicators. For the second round, a Likert five-point scale questionnaire was designed to evaluate the importance of the new index system. Experts rated each indicator on a scale of “Very Important”, “Important”, “Moderately Important”, “Less Important” and “Not Important” assigning scores of 5, 4, 3, 2, and 1, respectively. The mean scores, standard deviations, and CVs for the expert ratings were calculated.

All 15 experts returned valid questionnaires, achieving a 100% response rate. Table 3 and Table 4 show the average scores for the three primary indicators were above 3.5, with CVs all below 0.25. The Kendall coordination coefficient was W = 0.628; W = 0.628; W = 0.628, and the consistency test showed *p* < 0.001; *p* < 0.001; *p* < 0.001. The results for the secondary indicators revealed the average scores for all 11 secondary indicators were above 3.5, with coefficients of variation below 0.25. The Kendall coordination coefficient W = 0.443; W = 0.443; W = 0.443, and the consistency test showed *p* < 0.001; *p* < 0.001; *p* < 0.001. These results indicate a high level of consistency and agreement among experts for the primary and secondary indicators, meeting the standard requirements.

However, for the tertiary indicators, the results indicated that the coefficients of variation for B1.2 (Five-Level Frog Jumps) and C2.3 (1-Minute Double Under Jump Rope) were greater than 0.25, reflecting significant disagreement among experts. Consequently, these two indicators were deleted. In this round of expert consultation, some experts suggested that C2.1 (400 m×3) be changed to 400 m×5 to better reflect specificity, A2.1 (Arm Span) be changed to Arm Length, and B1 (Strength Quality) be changed to Relative Strength Quality. These suggestions were adopted. All other indicators met the screening criteria and required no modifications. 

### 3.4. Results of the Third Round of Expert Consultation

Building on the modifications made by experts in the second round, a third round of indicator consultation was conducted. All 15 experts returned valid questionnaires, achieving a 100% response rate. Table 5 and Table 6 show the average scores for the three primary indicators in this round were all greater than 3.5, with CVs all less than 0.25. The Kendall coordination coefficient W = 0.588; W = 0.588; W = 0.588, and the consistency test showed *p* < 0.001; *p* < 0.001; *p* < 0.001.

For the nine secondary indicators, the average scores were all greater than 3.5, with CVs all less than 0.25. The Kendall coordination coefficient was W = 0.465; W = 0.465; W = 0.465, and the consistency test showed *p* < 0.001; *p* < 0.001; *p* < 0.001. Based on these standards, the primary and secondary indicators met the requirements.

**Figure 4:**
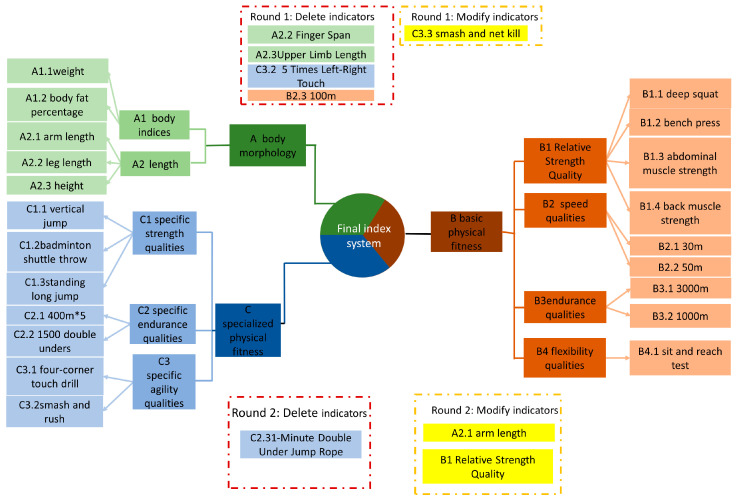
Physical fitness evaluation system for elite male singles badminton players.

For the 21 tertiary indicators in this round, the average scores were all greater than 3.5, with CVs all less than 0.25. The Kendall coordination coefficient was W = 0.471; W = 0.471; W = 0.471, and the consistency test showed *p* < 0.001; *p* < 0.001; *p* < 0.001. All tertiary indicators met the requirements, thus concluding the expert consultation phase. The final determined indicators are shown in Figure 4.

### 3.5. Establishing the Hierarchical Structure Model

**Figure 5:**
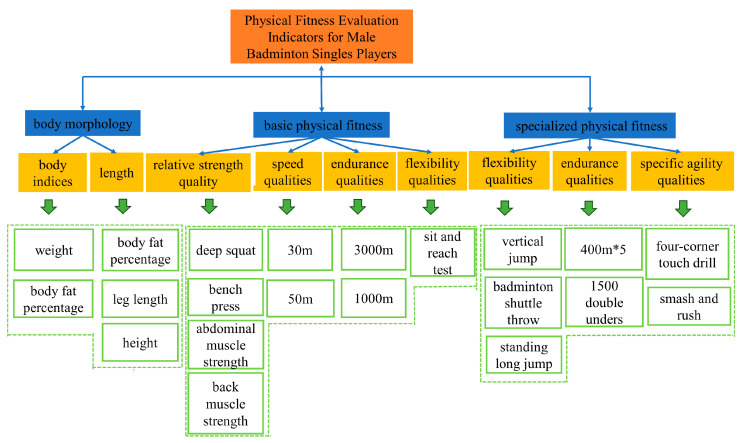
Hierarchical Structure Model.

In this study, “Physical Fitness Evaluation of Elite Male Singles Badminton Players” is set as the decision-making goal. The hierarchical structure model is constructed with “Body Morphology”, “Basic Physical Fitness” and “Specialized Physical Fitness” as the primary indicators, and “Body Index”, “Length”, “Relative Strength”, “Speed Quality”, “Endurance Quality”, “Flexibility Quality”, “Specialized Strength Quality”, “Specialized Endurance Quality” and “Specialized Agility Quality” as the secondary indicators. The 21 tertiary indicators serve as the bottom-layer elements. The hierarchical structure model is illustrated in Figure 5.

### 3.6. Calculation of Indicator System Weights

**Figure 6:**
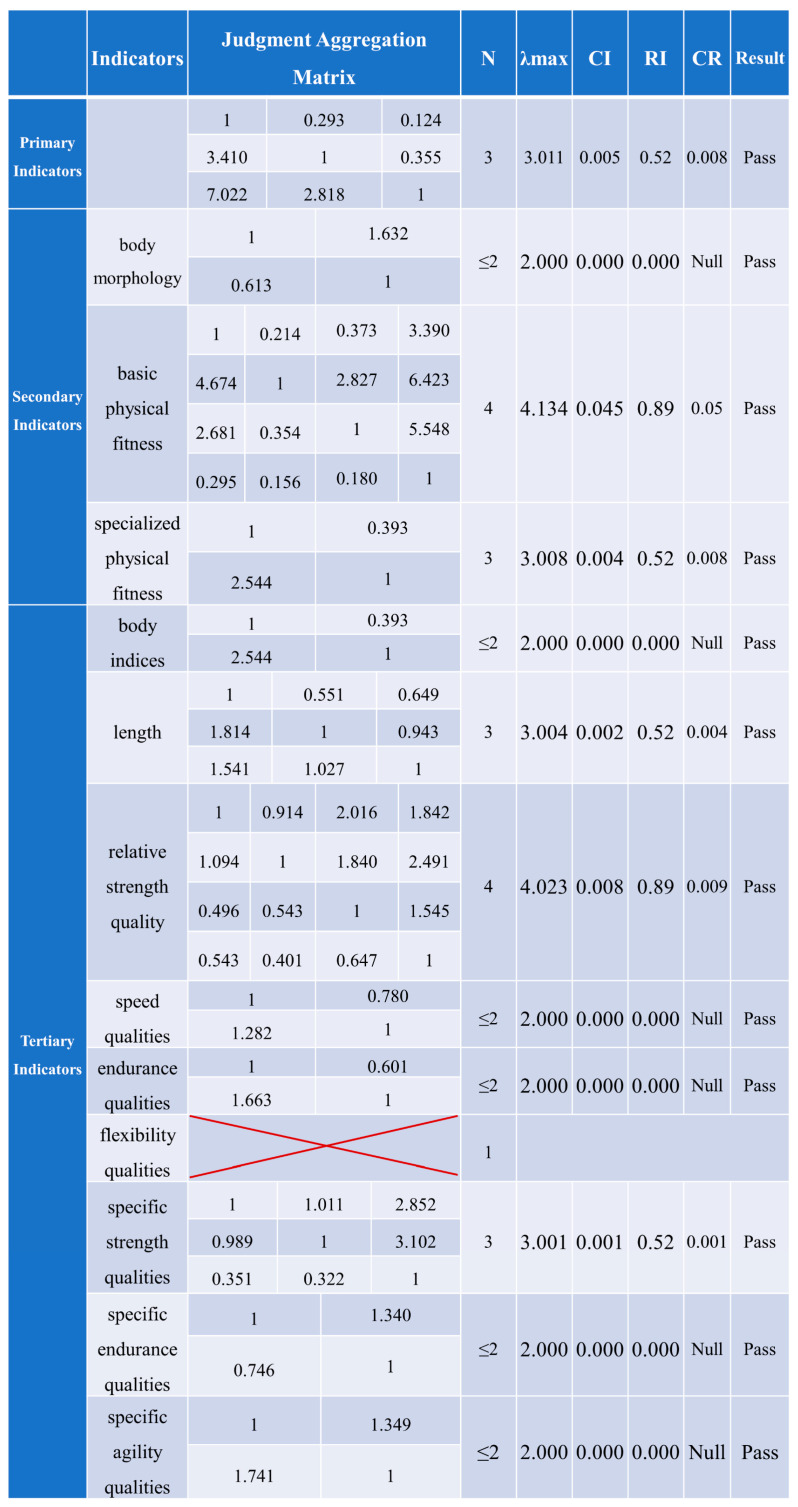
Consistency test results of indicators at each level.

**Figure 7:**
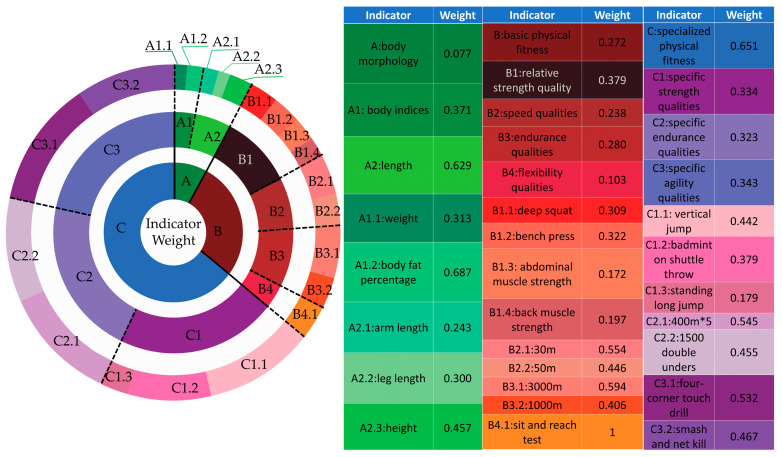
Weight of each indicator in the physical fitness evaluation system for elite male badminton singles players.

Figure 6 shows that the weight matrices provided by the experts passed the consistency test. The process involves first calculating the weights of the primary indicators, then the secondary indicators and tertiary indicators within the same level. Subsequently, the global weights of the secondary and tertiary indicators are calculated (Figure 7).

**Figure 8:**
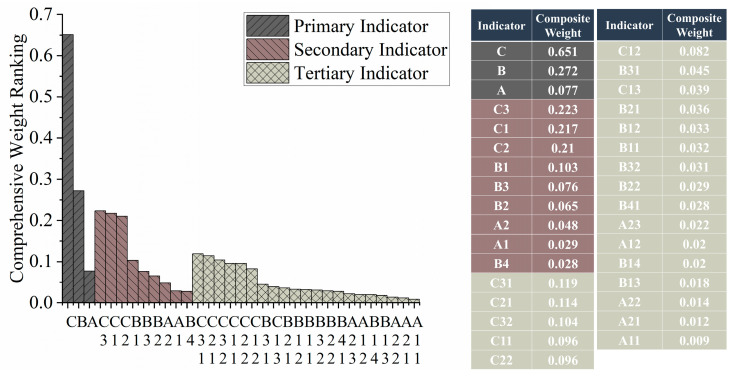
Comprehensive weight ranking of indicators at each level.

Comprehensive weight calculations and rankings for the indicators in the physical fitness evaluation system of elite male singles badminton players were conducted through the AHP (Figure 8). The results indicate that among the primary indicators, specialized physical fitness holds a dominant weight of 0.651, while Body Morphology has a smaller weight of 0.077. Among the secondary indicators, specialized agility, specialized strength, and specialized endurance have higher weights of 0.223, 0.217, and 0.210, respectively. Among the tertiary indicators, four-corner shuttle run, 400 m×5, smash and net kill, and vertical jump height have higher weights of 0.119, 0.114, 0.104, and 0.096, respectively.

These findings further emphasize the primary importance of specialized physical fitness, highlighting its decisive impact on an athlete’s performance. The high levels of agility, strength, and endurance suggest that athletes need comprehensive physical abilities to cope with various match situations. For coaches, the results provide clear directions and priorities for training. First, training should be designed based on the indicators to enhance athletes’ overall physical fitness. For instance, exercises like the four-corner shuttle run can improve agility and quick-response capabilities. Second, specialized strength training is essential, such as smash and net kill drills, to boost explosive power and hitting strength. Interval training and long-distance running, such as the 400 m×5 exercise, should be implemented to enhance cardiovascular function and endurance. The scientific design and implementation of these training contents will help comprehensively improve athletes’ competitive levels, laying a solid foundation for outstanding performance in matches.

### 3.7. Establishment and Application of the Evaluation System Standards

#### 3.7.1. Development of the Evaluation System Scoring Standards

The development of scoring standards aims to assign accurate numerical values to specific evaluation criteria to obtain fair, objective, and quantifiable evaluation results. The specific steps are as follows:

Data Collection: The average value, standard deviation, maximum value, and minimum value for each indicator were calculated to establish a general value model table for the physical fitness of elite male badminton players (Table 7).

This study used the percentile method to divide the collected data into 20 equal parts, with each part corresponding to 5 percentage points (Table 8 and Table 9). The percentile method is a widely used statistical analysis technique that effectively describes the value at a specific percentile position in the data distribution. Its main advantage is that it does not require the assumption that the data conform to a normal distribution, thereby eliminating the need for normality testing before setting the standards. Specifically, the percentile method involves sorting the data in ascending order and determining the data value at a specific percentile position to reflect the data distribution. For example, the 50 th percentile indicates that 50% of the data values are less than or equal to this value, and the remaining 50% are greater than or equal to it. This method uses the median and other percentiles as measures of dispersion, allowing for a categorized evaluation of the data and a graded assessment of the athletes’ performance levels. The percentile method is extensively used in various fields, both domestically and internationally, due to its simplicity and wide applicability. This method provides an intuitive description of data distribution and a solid foundation for establishing scientific and reasonable evaluation standards by analyzing data from large sample surveys [41,42]. It should be noted that eight indicators, Body Fat Percentage (A1.2), 30 m (B2.1), 50 m (B2.2), 3000 m (B3.1), 1000 m (B3.2), 400 m×5 (C2.1), Four-Corner Shuttle Run (C3.1), and Smash and Net Kill (C3.2), are in descending order, contrary to other indicators.

**Figure 9:**
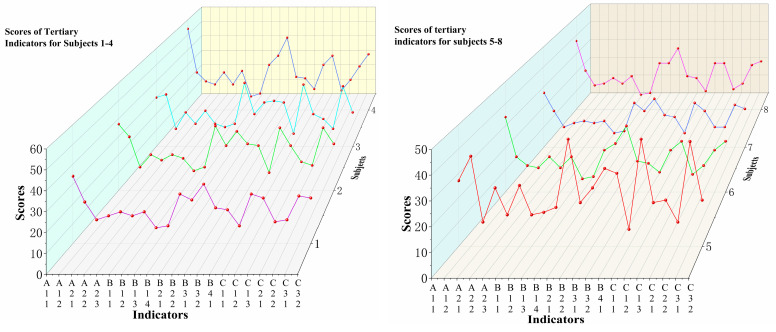

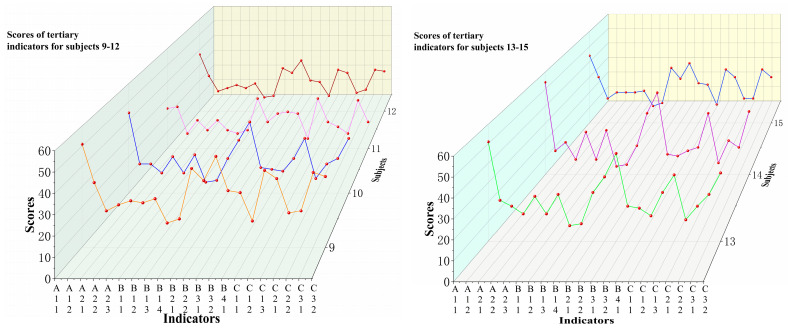
Weighted scores of tertiary indicators for physical fitness of elite male singles badminton players. Note: Different colors of lines represent the scoring conditions of different athletes.

**Figure 10:**
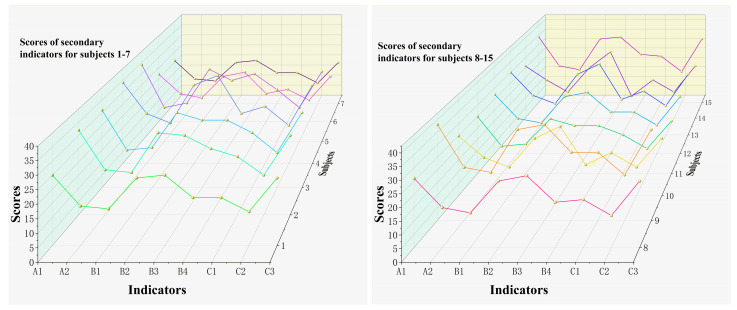
Weighted scores of secondary indicators for physical fitness of elite male singles badminton players. Note: Different colors of lines represent the scoring conditions of different athletes.

**Figure 11:**
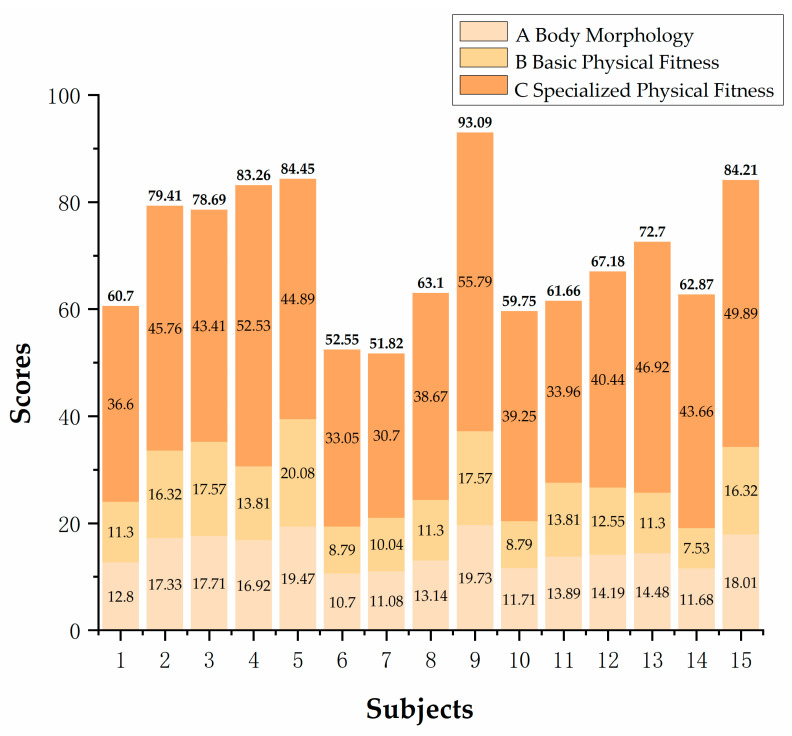
Weighted scores of primary indicators and total scores for physical fitness of elite male singles badminton players.

The comprehensive score results are obtained by combining individual scores with weights using the following calculation formula: $M=\sum m_{i}\beta_{i}$, where $m_{i}$ represents the score of each indicator, and $\beta_{i}$ represents the weight of each indicator. The calculation steps for the comprehensive evaluation results are as follows: Calculation of secondary indicator scores: Multiply the scores of each tertiary indicator by their respective weights and obtain their sum to determine the scores of each secondary indicator. Calculation of primary indicator scores: Multiply the scores of each secondary indicator by their respective weights and obtain their sum to determine the scores of each primary indicator. Calculation of comprehensive evaluation result: Multiply the scores of each primary indicator by their corresponding weights and obtain their sum to obtain the comprehensive evaluation result. The weighted scores for each level of indicators are shown in Figure 9, Figure 10 and Figure 11.

Based on the data above, a physical fitness level evaluation standard for elite male badminton players was established. The percentile method used in sports measurement and evaluation divides the rating into five levels: excellent, good, average, pass, and poor. The specific steps are as follows: Determine the levels: define the five levels as excellent, good, average, pass, and poor.

Calculate the threshold values: determine the actual measured values corresponding to each level’s threshold. Assign scores: assign different scores based on the interval divisions of the levels. List the results: tabulate the results to form a percentile-based rating table. This study uses the common rating division standard: above 90% is excellent, 75%–90% is good, 25%–75% is average, 10%–25% is pass, and below 10% is poor (Table 10). The level evaluation results for each primary indicator are shown in Table 11.

#### 3.7.2. Application of the Physical Fitness Evaluation System

This study randomly selected ten elite male singles badminton players from the China national badminton team to apply the established physical fitness evaluation system for analysis.

The athletes’ test data were entered into the physical fitness evaluation system for elite male badminton players, as shown in Table 12. The table shows that out of the 10 athletes, only three achieved an excellent rating in body morphology, four were rated as good, and three as average. In terms of basic physical fitness, five athletes were rated excellent, one good, and four average. For specialized physical fitness, six athletes achieved an excellent rating, one good rating, and three average ratings. Overall, four athletes were rated excellent, two good, and four average.

## 4. Discussion

The physical fitness evaluation index system for elite male badminton players, established through the Delphi method, selects body morphology, basic physical fitness, and specialized physical fitness as primary indicators. This system includes nine secondary indicators and twenty-one tertiary indicators (Figure 6). The aim is to comprehensively understand the physical fitness status of the athletes through systematic and scientific indicator evaluation, enabling the development of more targeted training programs to enhance their competitive performance and match outcomes.

Research has shown that body composition is an important indicator of physical fitness, and an athlete’s physique directly impacts their performance. By precisely managing body composition, athletes can not only improve their performance in the short term but also maintain good physical condition and health throughout their athletic careers. [24,25,26,43]. Different sports require different body morphology characteristics, which are particularly important for the biomechanical properties during physical activity [44,45]. Among the body morphology indicators, weight, body fat percentage, arm length, leg length, and height are included.

Weight and body fat percentage are crucial factors for evaluating an athlete’s physical fitness because excessive weight and body fat can significantly negatively impact athletic performance. Previous studies on badminton players have indicated a significant negative correlation between fat content and an athlete’s aerobic capacity. A higher body fat percentage can increase the athlete’s body temperature during exercise, making them more susceptible to fatigue and thereby reducing aerobic capacity. Additionally, it imposes an extra strain on heart function, which affects the efficiency and endurance of the cardiovascular system [46,47,48,49]. Excessive weight also means athletes bear more load during repeated jumps and quick movements, requiring them to have a higher capacity to carry their body weight [26,50]. These adverse factors can prevent athletes from maintaining optimal performance during high-intensity activities, thus affecting their overall performance and competitive level. Phoumsoupha et al. [51] pointed out that top-ranked badminton players are, on average, 5 cm taller than lower-ranked players, indicating that greater height might be an advantage, with taller players performing better. From a sports biomechanics perspective, arm length, leg length, and height provide significant mechanical leverage advantages [52]. Specifically, longer limbs can increase the length of the lever arm, thereby improving torque efficiency, which is crucial for quick movements and effective hitting in badminton. Longer arms and legs help athletes cover the court faster and hit the shuttlecock from higher positions, enhancing their offensive and defensive effectiveness [53]. These physical characteristics provide athletes with a significant performance advantage, allowing them to demonstrate higher efficiency and stability in quick responses and high-intensity movements during matches.

Badminton is considered the most demanding racquet sport in the world [51,54]. Athletes need to make quick movements and change directions as necessary, which undoubtedly requires them to possess excellent strength, speed, endurance, and flexibility [55]. Tiwari et al. [56] found a significant correlation between these qualities and athletes’ competitive abilities in studies of sub-elite athletes. Therefore, relative strength, speed, endurance, and flexibility were included in the basic physical fitness indicators. Relative strength was included with four tertiary indicators: deep squat, bench press, abdominal muscles, and back muscles. Deep squats and bench presses are essential indicators for assessing an athlete’s strength, reflecting the lower and upper body strength critical for jumping and hitting in badminton [21,57]. Evaluating the abdominal and back muscles helps understand the athlete’s core strength, which is crucial for maintaining body stability and power transfer. Quick movements, posture changes, hitting power, and accuracy in badminton require strong core strength and high dynamic balance [28,58].

The speed indicators of the 30- and 50-m runs evaluate short-distance explosive power and acceleration. Frequent quick movements and sudden stops are required in badminton matches, and these short-distance speed tests can accurately reflect the athlete’s acceleration and reaction capabilities, enhancing quick movements and positional changes in matches [51]. Badminton matches typically last between 40 min and 1 h [27,59]. The high intensity and frequency of matches require badminton players to have strong aerobic energy capacity [60,61]. The endurance indicators of the 3000- and 1000-m runs are chosen based on the high intensity and duration requirements of badminton matches to assess the athlete’s cardiovascular function and endurance level, ensuring they can maintain physical fitness during long, high-intensity matches. Flexibility plays a crucial role in badminton because it allows athletes to move their bodies and limbs to a wide range of motion within the limited space of the court. This flexibility is essential for effective returns and quick turns and affects an athlete’s agility, technical performance, and injury prevention [56,62].

For non-rhythmic sports such as badminton, handball, and basketball, traditional physical fitness testing methods using standard bicycles or treadmills are insufficient to accurately predict an athlete’s performance in competition [27]. Therefore, this study included specialized physical fitness as one of the primary indicators to ensure the evaluation results accurately reflect the physical demands and performance levels required in actual matches. Within the specialized physical fitness primary indicator, three secondary indicators were included: specialized strength, specialized endurance, and specialized agility. These indicators aim to assess the specific physical fitness requirements of athletes in badminton matches. Specialized Strength: The tertiary indicators of vertical jump, badminton throw, and standing long jump effectively assess the athlete’s explosive power and strength application ability, which are crucial for jumping and hitting actions [18,51,57,63]. Specialized Endurance: The 400 m×5 and 1-min double-under jump rope tests evaluate the athlete’s ability to sustain high-intensity activity throughout the match, ensuring they can maintain high performance levels during the later stages of the game [51,64,65]. Specialized Agility: Agility refers to the ability to rapidly change the speed or direction of the entire body in response to specific stimuli during movement [66]. The tertiary indicators of smash and net kill and the four-corner shuttle run assess the athlete’s quick reaction and agility, which often determine the outcome of a match in critical moments [67]. The inclusion of specialized physical fitness ensures that the evaluation system is comprehensive and tailored to the specific demands of badminton, providing a more accurate assessment of the athletes’ capabilities and performance potential in real match scenarios.

The evaluation results and test data of the ten athletes mentioned above in the aspects of body morphology, basic physical fitness, and specialized physical fitness were obtained, and a comprehensive assessment was conducted. The results indicate significant differences in the athletes’ performance across various indicators. Thus, this paper further discusses the implications of these results for coaches in terms of training plan formulation and execution, as well as for athletes’ training. From the coach’s perspective, the physical fitness evaluation results highlight certain issues in the formulation and execution of training plans. Coaches need to develop more targeted training plans based on the individual differences of athletes. For athletes who perform poorly on certain physical fitness indicators, training should focus on improving these weaknesses. Coaches should also enhance the monitoring and evaluation of athletes’ physical fitness changes, adjusting training content and intensity in a timely manner to ensure that athletes achieve optimal conditions in all aspects. From the athlete selection perspective, the selection process should not only focus on the technical level of the athletes but also comprehensively consider factors such as body morphology, basic physical fitness, and specialized physical fitness. This approach ensures the selection of truly promising athletes with development potential. For athletes whose physical fitness evaluation results are not ideal, coaches can improve their performance through targeted physical training. From the athletes’ training perspective, the results of the physical fitness evaluation system provide clear directions for their training. Athletes should recognize their strengths and weaknesses based on the evaluation results and make improvements in their daily training. For example, athletes with weaker body morphology should increase the proportion of basic and specialized physical fitness training. Conversely, athletes with poor specialized physical fitness should focus more on specialized technical and related physical training. Athletes can comprehensively improve their physical fitness levels through systematic and scientific training arrangements, laying a solid foundation for achieving excellent performance in competitions.

This study has several limitations. First, the research subjects are limited to elite male singles badminton players from China, which may constrain the applicability of the findings to athletes of different genders, countries, and regions. Although Chinese athletes hold a prominent position in international badminton, the generalizability of these results to other cultural and training environments has not been established. Second, the consulted experts are all from China, and their professional opinions may have regional and cultural biases, lacking a comprehensive inclusion of professional perspectives from diverse global backgrounds. This might limit the applicability of the evaluation index system on a worldwide scale. Additionally, since the study focuses on the characteristics of high-level athletes in China, the constructed physical fitness evaluation index system may not fully capture the needs and characteristics of athletes at other levels or backgrounds. Therefore, future research should consider broader samples and more diverse expert opinions to enhance the generalizability and scientific validity of the findings.

## 5. Research Limitations and Future Research Directions

Although this study has achieved certain findings and results, it presents several unavoidable limitations. The subjects were elite male singles badminton players at the national level in China, selected using stringent criteria, thus limiting the number of athletes who could participate. Future research should increase the sample size to enhance the representativeness and scientific validity of the results. Moreover, the reliance on data from Chinese elite players may limit the generalizability of the results. This regional restriction could affect the applicability of the evaluation system in other cultural contexts. Future studies are planned to include athletes from various countries to enhance the global applicability of the system. Another limitation is the lack of longitudinal data, which restricts demonstrating the long-term effectiveness of the system and its practical value in improving athletic performance. Future research should focus on collecting and analyzing longitudinal data, which would help to assess the ongoing impact of the evaluation system on long-term training and performance, as well as determine if adjustments are needed in the evaluation metrics or methods. Moreover, although the Delphi method reduced individual biases through collective consensus, it could not completely eliminate the subjectivity of expert opinions, which might influence the study results. Additionally, the absence of a control group is a clear limitation. Ideally, future studies would include non-elite athletes or those using different evaluation metrics to provide a broader comparison and validate the effectiveness of the evaluation system. Finally, the study’s focus on elite male singles players may limit the broader applicability of the findings. Including female athletes, doubles players, and athletes from other sports in future studies would enhance the universality and applicability of the evaluation system, helping to more fully understand and meet the diverse needs and characteristics of different athlete groups.

## 6. Conclusions

The established indicator system for evaluating the physical fitness of elite male singles badminton players comprises three primary indicators: “Body Morphology”, “Basic Physical Fitness” and “Specialized Physical Fitness”, alongside nine secondary and twenty-one tertiary indicators. The Analytic Hierarchy Process (AHP) results highlighted the predominance of specialized physical fitness among primary indicators. Secondary indicators such as specialized agility, strength, and endurance, along with tertiary indicators including four-corner shuttle runs, 400 m×5 shuttle runs, smash and net kill, and vertical jump height, were weighted significantly. This system’s application to elite badminton players validated its scientific accuracy and practical value, providing comprehensive fitness assessments that guide targeted training plans and optimize performance outcomes. The evaluation system and assigned weights proved to enhance the accuracy and objectivity of fitness assessments, demonstrating significant practical value in athlete training and development.

## Figures and Tables

**Table 1:** Summary of the Delphi expert group’s basic information.

Number	Name	Position
1	Wang *	National Team Coach
2	Wang *	National Team Coach
3	Xu *	First-line Coach
4	Ni *	First-line Coach
5	Fang *	First-line Coach
6	Chen*	First-line Coach
7	Ding *	First-line Coach
8	Jing *	First-line Coach
9	Lin *	Professor
10	Zhang *	Associate Professor
11	Wang *	Professor
12	Zhu *	Professor
13	Zhang *	Associate Professor
14	Chen *	Professor
15	Zheng *	Professor

Note: The “*” is used to protect the privacy of the consulting experts.

**Table 2:** Expert judgment basis coefficient assignment table.

Judgment Basis	The Impact of Criteria on Expert Judgment (Ca)		
	Significant Impact	Moderate Impact	Minor Impact
Practical experience	0.5	0.4	0.3
Logical reasoning	0.3	0.2	0.1
Research experience	0.1	0.1	0.1
Intuition	0.1	0.1	0.1
Aggregate	1.0	0.8	0.6

**Table 3:** Analysis parameters of indicators at each level in the second round.

**Primary Indicators**		
**Indicators**	**M ± SD**	**Cv**
A body morphology	3.93 ± 0.616	0.157
B basic physical fitness	4.36 ± 0.745	0.17
C specialized physical fitness	5.00 ± 0.000	0
**Secondary Indicators**		
**Indicators**	**M ± SD**	**Cv**
A1 body indices	4.36 ± 0.497	0.114
A2 length	4.57 ± 0.514	0.112
B1 strength qualities	4.36 ± 0.497	0.114
B2 speed qualities	4.43 ± 0.646	0.146
B3 endurance qualities	4.57 ± 0.514	0.112
B4 flexibility qualities	4.50 ± 0.519	0.115
C1 specific strength qualities	4.43 ± 0.514	0.116
C2 specific endurance qualities	4.64 ± 0.497	0.107
C3 specific agility qualities	4.86 ± 0.363	0.074
**Tertiary Indicators**		
**Indicators**	**M ± SD**	**Cv**
A1.1 weight	4.71 ± 0.469	0.1
A1.2 body fat percentage	4.64 ± 0.497	0.107
A2.1 arm span	4.50 ± 0.519	0.115
A2.2 leg length	4.57 ± 0.514	0.112
A2.3 height	4.71 ± 0.469	0.1
B1.1 deep squat	4.64 ± 0.497	0.107
B1.2 Five-Level Frog Jumps	2.50 ± 0.759	0.303
B1.3 bench press	4.79 ± 0.426	0.089
B1.4 abdominal muscle strength	4.71 ± 0.469	0.1
B1.5 back muscle strength	4.79 ± 0.426	0.089
B2.1 30 m	4.64 ± 0.497	0.107
B2.2 50 m	4.50 ± 0.519	0.115
B3.1 3000 m	4.79 ± 0.426	0.089
B3.2 1000 m	4.71 ± 0.469	0.1
B4.1 sit and reach test	4.79 ± 0.426	0.089
C1.1 vertical jump	4.79 ± 0.426	0.089
C1.2 badminton shuttle throw	4.53 ± 0.516	0.115
C1.3 standing long jump	4.60 ± 0.507	0.110
C2.1 400 m×3	4.57 ± 0.514	0.112
C2.2 1500 double unders	4.86 ± 0.363	0.075
C2.3 1-Minute Double Under Jump Rope	2.57 ± 0.646	0.251
C3.1 four-corner touch drill	4.79 ± 0.426	0.089
C3.2 Smash and Net Kill	4.64 ± 0.497	0.107

**Table 4:** Consistency test statistics of indicators at each level in the second round.

	Kendall (W)	*p*
Primary Indicators	0.628	<0.001
Secondary Indicators	0.443	<0.001
Tertiary Indicators	0.672	<0.001

**Table 5:** Analysis parameters of indicators at each level in the third round.

**Primary Indicators**		
**Indicators**	**M ± SD**	**Cv**
A body morphology	4.40 ± 0.507	0.11
B basic physical fitness	4.60 ± 0.507	0.11
C specialized physical fitness	5.00 ± 0.000	0
**Secondary Indicators**		
**Indicators**	**M ± SD**	**Cv**
A1 body indices	4.20 ± 0.676	0.161
A2 length	4.33 ± 0.617	0.142
B1 relative strength quality	4.40 ± 0.507	0.115
B2 speed qualities	4.40 ± 0.632	0.143
B3 endurance qualities	4.40 ± 0.737	0.167
B4 flexibility qualities	4.47 ± 0.516	0.115
C1 specific strength qualities	4.53 ± 0.516	0.114
C2 specific endurance qualities	4.40 ± 0.507	0.115
C3 specific agility qualities	4.60 ± 0.507	0.110
**Tertiary Indicators**		
**Indicators**	**M ± SD**	**Cv**
A1.1 weight	4.33 ± 0.816	0.188
A1.2 body fat percentage	4.47 ± 0.640	0.143
A2.1 arm length	4.33 ± 0.816	0.188
A2.2 leg length	4.40 ± 0.737	0.168
A2.3 height	4.47 ± 0.516	0.115
B1.1 deep squat	4.47 ± 0.743	0.166
B1.2 bench press	4.53 ± 0.516	0.113
B1.3 abdominal muscle strength	4.53 ± 0.516	0.113
B1.4 back muscle strength	4.27 ± 0.704	0.164
B2.1 30 m	4.27 ± 0.704	0.164
B2.2 50 m	4.33 ± 0.816	0.188
B3.1 3000 m	4.13 ± 0.743	0.180
B3.2 1000 m	4.33 ± 0.617	0.142
B4.1 sit and reach test	4.73 ± 0.594	0.126
C1.1 vertical jump	4.67 ± 0.488	0.104
C1.2 badminton shuttle throw	4.80 ± 0.414	0.086
C1.3 standing long jump	4.73 ± 0.458	0.097
C2.1 400 m×5	4.20 ± 0.676	0.161
C2.2 1500 double unders	4.33 ± 0.617	0.142
C3.1 four-corner touch drill	4.40 ± 0.507	0.115
C3.2 smash and net kill	4.79 ± 0.426	0.089

**Table 6:** Consistency test statistics of indicators at each level in the third round.

	Kendall (W)	*p*
Primary Indicators	0.588	<0.001
Secondary Indicators	0.465	<0.001
Tertiary Indicators	0.471	<0.001

**Table 7:** General measurement model for physical fitness of elite male badminton singles players.

Test Indicator	Unit	Minimum	Maximum	Average	SD
A1.1 weight	kg	65.00	90.00	72.94	4.07
A1.2 body fat percentage	%	10.00	19.00	13.69	2.82
A2.1 arm length	cm	52.00	67.00	58.66	4.07
A2.2 leg length	cm	102.00	118.00	108.22	4.74
A2.3 height	cm	175.00	193.00	182.00	4.25
B1.1 deep squat	Kg/BW	1.33	2	1.68	0.23
B1.2 bench press	Kg/BW	0.73	1.4	1.01	0.20
B1.3 abdominal muscle strength	min	6.85	7.33	2.36	0.82
B1.4 back muscle strength	min	2.04	3.09	2.24	0.34
B2.1 30 m	s	3.81	4.57	4.13	0.22
B2.2 50 m	s	6.61	8.25	7.31	0.49
B3.1 3000 m	s	611.00	768.00	678.47	47.02
B3.2 1000 m	s	190	270	208	11.67
B4.1 sit and reach test	cm	10.00	26.00	15.25	4.45
C1.1 vertical jump	cm	59.00	71.00	65.75	2.91
C1.2 badminton shuttle throw	cm	595	789	697	63.54
C1.3 standing long jump	cm	269	286	276.06	5.03
C2.1 400 m×5	s	55.00	70.00	66.38	3.38
C2.2 1500 double unders	min	13.13	17.30	15.62	1.13
C3.1 four-corner touch drill	s	29.00	32.68	31.22	0.91
C3.2 smash and net kill	s	30.00	36.11	32.20	1.30

Note: BW = body weight.

**Table 8:** Performance rating criteria for individual physical fitness indicators of elite male badminton singles players.

Score	A1.1	A1.2	A2.1	A2.2	A2.3	B1.1	B1.2	B1.3	B1.4	B2.1	B2.2
100	100	7	80	132	198	2.3	1.3	7.42	5.58	3.74	6.52
95	98	8	78	130	196.64	2.24	1.26	7.39	5.56	3.76	6.55
90	95	9	76	128	195.28	2.18	1.22	7.36	5.54	3.79	6.58
85	92	10	74	126	193.92	2.12	1.18	7.33	5.52	3.82	6.61
80	89	11	72	124	192.56	2.06	1.14	7.30	5.50	3.85	6.64
75	85	12	70	122	191.2	2	1.1	7.27	5.48	3.88	6.67
70	84	13	68	120	189.84	1.94	1.06	7.24	5.46	3.91	6.70
65	83	14	66	118	188.48	1.88	1.02	7.21	5.44	3.94	6.74
60	81	15	64	116	187.12	1.82	0.98	7.18	5.42	3.97	6.77
55	80	16	62	114	185.76	1.76	0.94	7.15	5.40	4.00	6.80
50	78	17	60	112	184.4	1.7	0.9	7.12	5.38	4.03	6.83
45	75	18	58	110	183.04	1.64	0.86	7.09	5.36	4.06	6.87
40	73	19	56	108	181.68	1.58	0.82	7.06	5.34	4.09	6.90
35	71	20	54	106	180.32	1.52	0.78	7.03	5.32	4.12	6.93
30	68	21	52	104	178.96	1.46	0.74	7.00	5.30	4.15	6.96
25	65	22	50	102	177.6	1.4	0.7	6.93	5.28	4.18	6.99
20	62	23	48	100	176.24	1.34	0.66	6.90	5.26	4.21	7.02
15	60	24	46	98	174.88	1.28	0.62	6.87	5.24	4.25	7.05
10	58	25	44	96	173.52	1.22	0.58	6.85	5.22	4.27	7.08
5	56	26	42	94	172.16	1.16	0.54	6.82	5.20	4.30	7.71
0	53	27	40	92	170.75	1.1	0.5	6.79	5.18	4.33	7.74

**Table 9:** Performance Rating Criteria for Individual Specialized Physical Fitness Indicators of Elite Male Badminton Singles Players.

Score	B3.1	B3.2	B4.1	C1.1	C1.2	C1.3	C2.1	C2.2	C3.1	C3.2
100	600	180	28	100	950	330	42	27	23	27
95	610	185	27	97	930	325	44	26	23.5	27.5
90	620	190	26	94	910	320	46	25	24	28
85	630	195	25	91	890	315	48	24	24.5	28.5
80	640	200	24	88	870	310	50	23	25	29
75	650	205	23	85	850	305	52	22	25.5	29.5
70	660	210	22	82	830	300	54	21	26	30
65	670	215	21	79	810	295	56	20	26.5	31.5
60	680	220	20	76	790	290	58	19	27	32
55	690	225	19	73	770	285	60	18	27.5	32.5
50	700	230	18	70	750	280	62	17	28	33
45	710	235	17	67	730	275	64	16	28.5	33.5
40	720	240	16	64	710	270	66	15	29	34
35	730	245	15	61	690	265	68	14	29.5	34.5
30	740	250	14	58	670	260	70	13	30	35
25	750	255	13	55	650	255	72	12	31.5	35.5
20	760	260	12	52	630	250	74	11	32	36
15	770	265	11	49	610	245	76	10	32.5	36.5
10	780	270	10	46	590	240	78	9	33	37
5	790	275	9	43	570	235	80	8	33.5	37.5
0	800	280	8	40	550	230	82	7	34	38

**Table 10:** Percentile method grading standards.

Evaluation Grade	Standard	Percentage
Excellent	≥90%	10%
Good	75% (incl.)~90%	15%
Average	25% (incl.)~75%	50%
Pass	10%~25%	15%
Poor	≤10%	10%

**Table 11:** Special physical fitness evaluation standards for elite male badminton singles players.

	Poor	Pass	Average	Good	Excellent
Body Morphology	≤6.42	6.43–12.42	12.43–24.14	24.15–30.5	≥30.55
Basic Physical Fitness	≤8.36	8.37–15.77	15.78–28.94	28.95–40.14	≥40.15
Specialized Physical Fitness	≤14.61	14.62–22.84	22.85–40.75	40.76–54.72	≥54.73

**Table 12:** Evaluation grade results for special physical fitness of elite male badminton singles players.

Subjects	Body Morphology	Basic Physical Fitness	Specialized Physical Fitness	Overall Situation
N1	Good	Excellent	Excellent	Good
N2	Excellent	Excellent	Excellent	Excellent
N3	Average	Excellent	Average	Average
N4	Good	Excellent	Excellent	Excellent
N5	Average	Average	Average	Average
N6	Excellent	Average	Excellent	Excellent
N7	Good	Average	Average	Average
N8	Excellent	Good	Good	Good
N9	Average	Excellent	Excellent	Excellent
N10	Good	Average	Excellent	Average

## Data Availability

Data will be made available on request.
